# Rabies in Endangered Ethiopian Wolves

**DOI:** 10.3201/eid1012.040080

**Published:** 2004-12

**Authors:** Deborah A. Randall, Stuart D. Williams, Ivan V. Kuzmin, Charles E. Rupprecht, Lucy A. Tallents, Zelealem Tefera, Kifle Argaw, Fekadu Shiferaw, Darryn L. Knobel, Claudio Sillero-Zubiri, M. Karen Laurenson

**Affiliations:** * University of Oxford, Oxford, United Kingdom;; † Ethiopian Wolf Conservation Programme, Addis Ababa, Ethiopia;; ‡ Centers for Disease Control and Prevention, Atlanta, Georgia, USA,; § Ethiopian Wildlife Conservation Organisation, Addis Ababa, Ethiopia;; ¶ University of Edinburgh, Edinburgh, United Kingdom;; # Frankfurt Zoological Society, Arusha, Tanzania

**Keywords:** Canis simensis, endangered species, epidemic, Ethiopian wolves, rabies, wildlife diseases, dispatch

## Abstract

With rabies emerging as a particular threat to wild canids, we report on a rabies outbreak in a subpopulation of endangered Ethiopian wolves in the Bale Mountains, Ethiopia, in 2003 and 2004. Parenteral vaccination of wolves was used to manage the outbreak.

During the last decade, infectious diseases have posed a major risk to small populations of wild vertebrates. Highly pathogenic infectious agents have been implicated in the decline and extirpation of a considerable number of populations (*1**–**3*). Analysis of disease outbreaks suggests that carnivores appear to be particularly susceptible (*2*). Specifically, the susceptibility of wild canids may arise from a variety of intrinsic social and ecologic factors but is undoubtedly also due to their susceptibility to general pathogens carried by the most abundant carnivore, the domestic dog. Indeed, rabies has emerged as the most common cause of disease outbreaks in wild canids (*1**–**3*).

## The Outbreak

**Figure 1:**
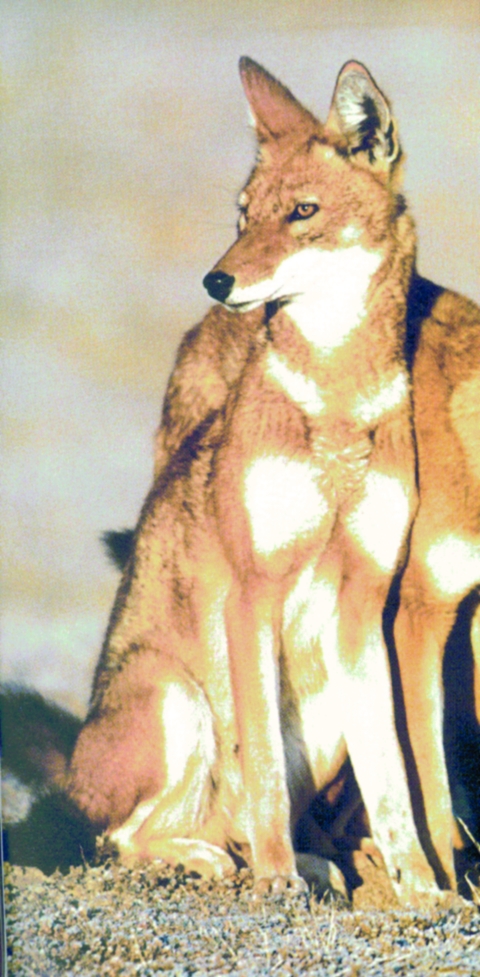
Ethiopian wolves. Photo credit: Martin Harvey.

**Figure 2:**
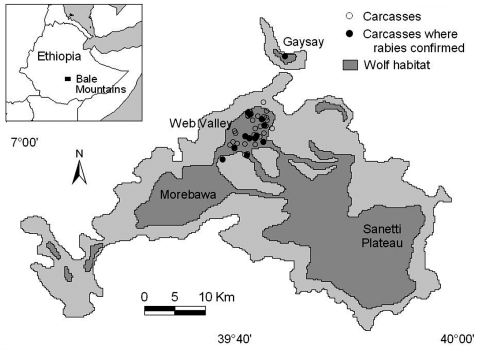
Ethiopian wolf subpopulations, habitat and carcass locations during the reported rabies outbreak in the Bale Mountains, Ethiopia. Samples were not obtained from all carcasses, but those confirmed rabies positive are depicted with filled circles.

We report on an outbreak caused by rabies in the world's rarest canid, the endangered Ethiopian wolf (*Canis simensis*) (Figure 1). The Ethiopian wolf (13–20 kg) is found in only seven Afroalpine highlands in Ethiopia. Wolves live in discrete and cohesive social packs of 2 to 18 adults that communally share and defend an exclusive territory but forage alone for small prey, primarily rodents. Breeding is usually monopolized by a dominant pair, although subordinate animals do attempt to breed, and all animals help raise young. With up to 300 of the global estimate of 500 wolves, the Bale Mountains in south-central Ethiopia are home to the largest and most important population of this species (*4*). Within these mountains, three areas of relatively high density of Ethiopian wolves can be identified (Figure 2), although wolves are found through the Afroalpine range. Until August 2003, one of these core areas, the Web Valley, harbored an estimated 95 wolves.

Wolf packs in the Bale Mountains are monitored by the Ethiopian Wolf Conservation Programme staff on foot or horseback, using binoculars, global positioning systems, and, following the rabies control intervention strategy, radio telemetry. Over the last year, the Ethiopian Wolf Conservation Programme monitored 47 packs containing 250–300 wolves, in seven areas in these mountains. During a 6-week period from mid-August to the end of September, the carcasses of four wolves were found in the Web Valley; 15 more dead wolves were found in the first half of October (Table). Through January 30, 2004, a total of 38 wolf carcasses were found in this core monitoring area. Two carcasses were also found in the Morebawa to the south and one in the Gaysay Valley to the north (Figure 2). In addition, 36 wolves disappeared from the Web Valley during this period. Extrapolating from background annual average probability of death (0.15 for adults and yearlings, 0.45 and 0.55 for juveniles males and females, respectively), we would normally have expected ≈12 of 95 animals in the Web Valley subpopulation to die during these 6 months, rather than the 74 that actually died or disappeared. Thus, mortality clearly increased in this period. Clinical signs observed in 10 Ethiopian wolves (including 2–5 animals whose carcasses were not recovered) during this period were all consistent with rabies and included hind limb ataxia (8 wolves, including 3 that were repeatedly falling), depression (n = 6), severe weight loss (n = 3), restlessness or unusual ranging behavior (n = 4), aggression (n = 3), loss of fear of humans or other wolves (n = 2), excessive salivation at death (n = 2), and a poor coat (n = 1).

**Table:** Ethiopian wolf carcasses found from August 2003 through January 2004 in the Web Valley of the Bale Mountains, Ethiopia

Date interval	No. carcasses found	Cumulative total
Aug 11–24, 2003	1	1
Aug 25 to Sept 7, 2003	0	1
Sept 8–21, 2003	1	2
Sept 22–Oct 5, 2003	2	4
Oct 6–19, 2003	16	20
Oct 20–Nov 2, 2003	6	26
Nov 3–16, 2003	2	28
Nov 17–30, 2003	6	34
Dec 1–14, 2003	1	35
Dec 15–28, 2003	1	36
Dec 29, 2003, to Jan 11, 2004	1	37
Jan 12–25, 2004	0	37
Jan 26–Feb 8, 2004	1	38

**Figure 3:**
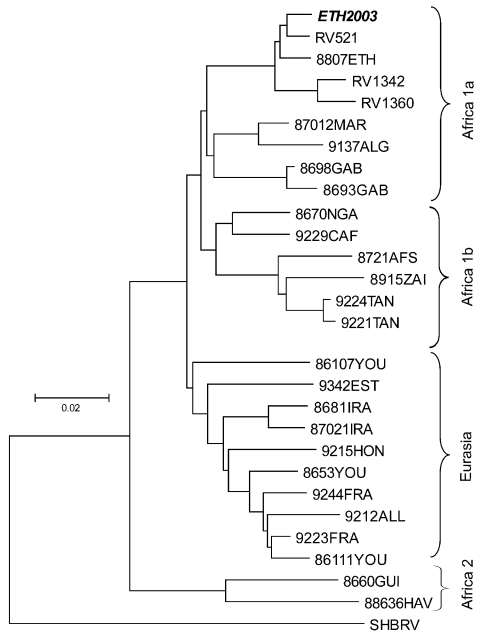
Neighbor-joining phylogenetic tree of African and Eurasian rabies virus samples, rooted with silver-haired bat rabies virus variant (SHBRV), based on a 400–base pair region of the nucleoprotein gene. The sample names are given according to GenBank records.

Rabies virus was diagnosed from 13 of 15 brain samples sent to the Centers for Disease Control and Prevention (CDC), USA. The negative samples most likely represent poor-quality samples, but these results are also consistent with background mortality. Diagnostics, RNA extraction from wolf brains, reverse transcription–polymerase chain reaction, N gene sequence generation, and phylogenetic analysis were performed as described in Kuzmin et al. (*5*). The nucleoprotein (N) gene sequences (strain ETH2003, GenBank accession no. AY500827) were identical for all 13 samples, which suggests a single point source of infection. Comparison with other N gene sequences available from GenBank (Figure 3) and limited sequences from the national CDC archival collection (to be submitted, GenBank accession nos. requested) demonstrated that the virus belonged to the Africa-1a group (*6*). The N gene of the virus ETH2003 was 98.3% identical to the N gene of the virus 8807ETH, isolated from an Ethiopian hyena (*Crocuta crocuta*) in 1987 but, in general, belongs to the overall group associated with domestic dogs in different regions throughout northern and central Africa (*6*). Overall identity in the group was 96.2% for the N gene sequences.

At least one previous rabies epidemic has occurred in the Bale Mountains: 77% of 53 known wolves died or disappeared in the Web Valley from 1991 to 1992 (*7*). Rabies was confirmed in samples from three animals (*7**,**8*). In addition, 52% of 23 known wolves died or disappeared in the Sanetti Plateau (Figure 2) from April to June 1990, but no samples were obtained for analysis (*7*). However, disease was identified as the prime candidate for this sudden reduction in numbers. Overall, the Bale Mountains wolf population was estimated to have declined from ≈450 to 120–160 animals in the early 1990s (*9*). There were, therefore, concerns that the recent outbreak would again spread throughout the whole Bale wolf population.

All available evidence suggests that domestic dogs are the reservoir for rabies both in the Bale Mountains and Ethiopia; the genetic analysis identified the virus to be of canid type and no wildlife reservoir has ever been identified in this country. Rabies is endemic in Ethiopia and remains both a public health and economic problem through livestock losses in these impoverished rural communities (*10**,**11*). More than 32 domestic dogs and 20 cattle exhibiting clinical signs consistent with rabies were reported in communities adjacent to the Bale Mountains National Park in the same period, and at least three people were bitten by suspected rabid dogs. Efforts have been made to reduce the threat of rabies to Ethiopian wolves in this area since 1996 through the vaccination of dogs. More than 70% of domestic dogs in core wolf areas within the national park have been vaccinated against rabies, and, where resources have allowed, the dog vaccination campaign has been extended to surrounding communities. Case traceback in this outbreak suggests rabies may have been brought into wolf habitat by an unvaccinated immigrant dog accompanying people and livestock searching for seasonal grazing.

Rabies was confirmed on October 28, 2003, and advice on its management was sought from a range of persons and institutions including the World Conservation Union/Species Survival Commission Canid Specialist Group and Veterinary Specialist Group. After recommendations were submitted, the Ethiopian Wildlife Conservation Organization decided to intervene with a trial emergency measure, on the grounds that that the species is rare and endangered and that rabies was apparently inadvertently introduced as a byproduct of human activities. The Ethiopian Wolf Conservation Programme implemented this trial intervention, which aimed to contain the disease within the area of the initial outbreak. Since oral rabies vaccines are not currently licensed for use in Ethiopia, parenteral vaccination was the only means to directly protect wolves. Wolf trapping and vaccination began in mid-November in packs adjacent to those already affected, and 69 wolves were trapped and vaccinated up to the middle of February in two high-density wolf areas, Sanetti (n = 33) and Morebawa (n = 36), outside of the core rabies-affected area. Wolves were injected intramuscularly with alternate 1-mL or 2-mL doses of inactivated rabies vaccine (Nobivac R, Intervet). A subsample of 19 wolves was recaptured 30 days later (±5 days) to determine the extent of antibody response to vaccination and to administer a booster dose of 1 mL of vaccine. Preliminary results have shown that all these 19 wolves seroconverted after vaccination. All but one vaccinated wolf were confirmed alive 1 week after vaccination, and all but two wolves were alive 2 months later; both figures are consistent with background death rates. A full analysis of this ongoing work will be reported when data are complete.

This outbreak has highlighted that rabies is a continued threat to endangered canids and that conservationists are often ill-equipped to manage infectious disease. First, lack of information often hinders management: few established models offer guidance. Indeed, some early and unsurprising failures have attracted damaging controversy (*2*). In addition, although a variety of theoretic approaches to disease management exist, relatively few may be feasible or effective (*12*). Finally, considerable funds are required to effectively prevent and control disease threats. Nevertheless, results obtained from this trial intervention will be invaluable in assessing the effectiveness of vaccination against rabies in Ethiopian wolves and of the approach in general. These findings will be particularly useful when considering disease management options for wolf populations in other areas of Ethiopia (*12**,**13*). With rabies-endemic dog populations around all Ethiopian wolf populations, further research and trials are required to ascertain the most cost-effective and feasible method to decrease the threat of disease for each population and to control any future outbreaks.
